# PARP1 suppression by α7 nAChR activation attenuated α-synuclein-induced neurotoxicity in Parkinson’s disease

**DOI:** 10.1093/procel/pwag004

**Published:** 2026-04-11

**Authors:** Xiaoxi Ren, Dandan Guan, Fenqin Xue, Feilong Zhang, Jing Sun, Yan Zheng, Haixia Huang, Zhi-Qing David Xu, Jianliang Zhang, Wei Wang, Chen Zhang

**Affiliations:** Department of Neurobiology, School of Basic Medical Sciences, Beijing Key Laboratory of Neural Regeneration and Repair, Beijing Institute of Brain Disorders, Capital Medical University, Beijing 100069, China; Laboratory for Clinical Medicine, Capital Medical University, Beijing 100069, China; Department of Pathology, School of Basic Medical Sciences, Capital Medical University, Beijing 100069, China; Department of Pathology, School of Basic Medical Sciences, Capital Medical University, Beijing 100069, China; Core Facilities Center, Capital Medical University, Beijing 100069, China; Department of Neurobiology, School of Basic Medical Sciences, Beijing Key Laboratory of Neural Regeneration and Repair, Beijing Institute of Brain Disorders, Capital Medical University, Beijing 100069, China; Department of Pathology, School of Basic Medical Sciences, Capital Medical University, Beijing 100069, China; Department of Physiology and Pathophysiology, Capital Medical University, Beijing 100069, China; Laboratory for Clinical Medicine, Capital Medical University, Beijing 100069, China; Department of Physiology and Pathophysiology, Capital Medical University, Beijing 100069, China; Department of Pathology, School of Basic Medical Sciences, Capital Medical University, Beijing 100069, China; Department of Neurobiology, School of Basic Medical Sciences, Beijing Key Laboratory of Neural Regeneration and Repair, Beijing Institute of Brain Disorders, Capital Medical University, Beijing 100069, China; Laboratory for Clinical Medicine, Capital Medical University, Beijing 100069, China; Department of Physiology and Pathophysiology, Capital Medical University, Beijing 100069, China; Department of Neurobiology, School of Basic Medical Sciences, Beijing Key Laboratory of Neural Regeneration and Repair, Beijing Institute of Brain Disorders, Capital Medical University, Beijing 100069, China

**Dear Editor,** 

Parkinson’s disease (PD) is the second most common neurodegenerative disease, and recent studies have suggested alpha-synuclein (α-syn) aggregates could induce pathological hyperactivation of poly(ADP-ribose) polymerase 1 (PARP1) (Kam et al., 2018), driving excessive PAR biosynthesis. Particularly, PAR might directly bind to α-syn, accelerating its fibrillization and converting α-syn aggregates to a more misfolded compact strain with enhanced toxicity (Kam et al., 2018), resulting in a further worsening of cell death (Park et al., 2022). These findings positioned the α-syn-PARP1 axis as a compelling therapeutic target for PD.

A tobacco component, nicotine, is believed to exert neuroprotective effects in PD by acting on nicotinic receptors (nAChRs) (Perez, 2015; Quik et al., 2014), although its underlying mechanism is still illusive. Among nAChRs, α7 nAChRs are widely expressed throughout the mammalian brain (Quik et al., 2015) and have been implicated in the regulation of proteins important for cell survival or synaptic plasticity (Dineley et al., 2015; Xu et al., 2021). Given the pivotal role of PARP1 in α-syn-driven neurodegeneration, research on suppression of PARP1 by stimulating α7 nAChR is crucial for developing disease-modifying therapies for PD.

To determine the effect of nicotine on α-syn-induced PARP1 hyperactivation, the PAR-conjugated proteins in primary neurons treated with α-syn and preformed fibril were measured. It showed α-syn PFF treatment led to a robust increase in the PAR-conjugated protein level (3–10 days post-treatment), which was prevented by nicotine pretreatment (Figs. 1A, 1B, and S1A). Furthermore, propidium iodide (PI) staining and cell viability assays further demonstrated that α-syn PFF-induced neuronal death and activity loss were significantly mitigated by both nicotine and the PARP1 inhibitor ABT-888, and their combination did not confer any additive effect, implying that nicotine might exert its neuroprotective effects via inhibiting PARP1 activation (Fig. S1B–D). To determine whether the neuroprotective effect of nicotine was mediated through α7 nAChRs, we employed d-tubocurarine (d-TC), a competitive orthosteric antagonist that displaces nicotine by binding the canonical agonist site, and methyllycaconitine citrate (MLA), a highly selective antagonist that binds to the orthosteric site of α7 nAChRs; both antagonists significantly inhibited nicotine’s protection against α-syn PFF toxicity (Fig. S1E–G), revealing that nicotine may play a neuroprotective role through α7 nAChRs. To reconsolidate this finding, the role of α7 nAChR was further tested at both pharmacological and genetic levels. Pharmacological assay revealed that pre-treatment with PNU282987, a selective α7 nAChR agonist that binds competitively to the canonical agonist site, recapitulated nicotine-mediated neuroprotection effect (Fig. S1E–H). Furthermore, both PNU282987 and nicotine reduced phosphorylated α-syn (p-α-syn, a marker of pathologic α-syn) and TX-100-insoluble α-syn level in neuronal cultures (Figs. 1C–D and S1I–L), indicating α7 nAChR activation inhibits α-syn pathology. Genetically, when α7 nAChRs were knocked down via short hairpin RNA (shRNA), the protective effects conferred by PNU282987 or nicotine were significantly abolished (Figs. 1E and S1M–O). Collectively, these data demonstrated activation of α7 nAChRs prevented α-syn PFF-induced PARP1 hyperactivation and inhibited α-syn neurotoxicity in neuronal cultures.

**Figure 1.**
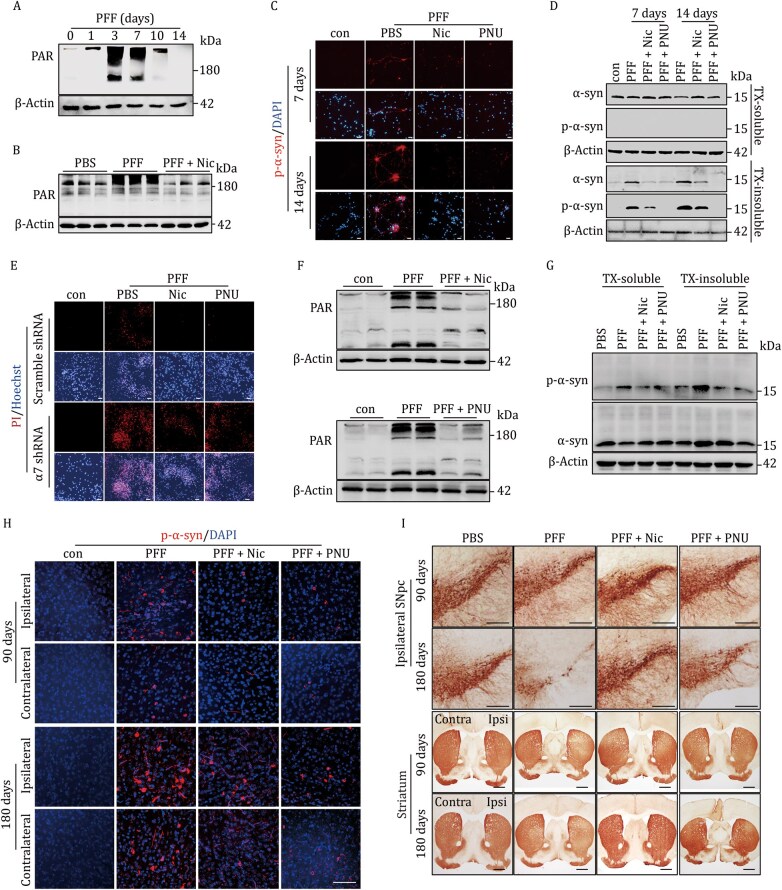
**Suppression of PARP1 hyperactivation by stimulation of α7 nAChRs inhibited α-syn neurotoxicity and rescued α-syn PFF-induced neurodegeneration *in vitro* and *in vivo***. (A) Activation of PARP1 in PFF-treated primary cortical neurons. (B) Primary cortical neurons were preincubated with nicotine and further incubated with PFF for 7 days. The PAR level was determined by western blot. (C) Representative images of p-α-syn immunostaining in primary cortical neurons treated with PFF, PFF + nicotine or PFF + PNU282987 for 7 or 14 days (*n* = 3). Scale bar, 20 µm. (D) Representative immunoblots of α-syn and p-α-syn in detergent-soluble and insoluble fractions from primary cortical neurons treated with PFF, PFF + nicotine or PFF + PNU282987 for 7 or 14 days (*n* = 3). (E) Representative images of Hoechst and PI staining of primary cortical neurons transduced with lentiviruses containing scramble shRNA control or α7 nAChR shRNA and further incubated with PFF for 14 days. Scale bar, 20 µm. (F) Representative PAR immunostaining in the striatum of mice at 90 days after PBS, PFF, PFF + nicotine or PFF + PNU282987 treatment (*n* = 3). (G) Representative immunoblots of midbrain lysates from PFF–, PFF+ nicotine-, or PFF + PNU282987-treated mice with α-syn, p-α-syn and β-actin antibodies (*n* = 3). (H) Representative p-α-syn immunostaining in the cortex of mice at 90 and 180 days after PBS, PFF, PFF + nicotine or PFF + PNU282987 treatment (*n* = 3). Scale bar, 75 µm. (I) Representative TH staining of SNpc DA neurons (scale bar, 400 µm) and striatal dopaminergic terminals (scale bar, 200 µm) of mice at 90 and 180 days after α7 nAChR agonist exposure and instrastriatal α-syn PFF injection.

Next, we determined whether α7 nAChR could ameliorate PARP1 hyperactivation-induced dopaminergic pathology following the intrastriatal α-syn PFF injection in mice. At 90 days post-injection, α-syn PFF injection significantly increased PAR levels and phosphorylated α-syn aggregates, both of which were suppressed by PNU282987 and nicotine (Figs. 1F–H and S2A–C). Furthermore, the immunohistochemical analysis showed α-syn PFF induced dopaminergic neuron loss in the substantia nigra pars compacta (SNpc) and striatal dendrite density reduction, which were markedly attenuated by stimulation of α7 nAChR (Figs. 1I and S2D–E). Behaviorally, PNU282987 and nicotine rescued α-syn PFF-induced deficits in grip strength (wire hang test) and motor coordination (rotarod assay) at multiple time points (Fig. S2F–G), confirming α7 nAChRs may play a potential role in inhibiting PARP1 hyperactivation and consequently rescuing α-syn PFF-induced neurodegeneration *in vivo*.

To further validate our findings, we investigated whether α7 nAChR activation could suppress PARP1 overactivation induced by MNNG (N-methyl-N′-nitro-N-nitrosoguanidine), a DNA-alkylating agent known primarily for causing DNA damage and potentially leading to PARP1 hyperactivation in certain cell types. The results demonstrated that α7 nAChR stimulation also significantly attenuated MNNG-induced PARP1 overactivation (Fig. S2H and S2I), directly supporting the inhibitory role of α7 nAChR in regulating PARP1.

Next, we explored the mechanism by which PARP1 was suppressed by α7 nAChRs. We first measured PARP1 protein level in primary neurons after stimulation of α7 nAChR, and observed no significant difference in PARP1 protein levels between various dose points (Fig. S3A) or treatment time points (Figs. 2A and S3B), regardless of stimulation with PNU282987 or nicotine. It has been reported that the sirt6 protein level is elevated in PD patient brains (Nicholatos et al., 2018), and sirt6 may physically associate with and mono-ADP-ribosylate PARP1, thereby stimulating PARP1 poly-ADP-ribosylase activity (Kong et al., 2020; Mao et al., 2011). Therefore, we hypothesized that α7 nAChR might suppress PARP1 activity by downregulating sirt6 protein levels. To test this hypothesis, we first treated primary neurons (Fig. S3C–E) and SH-SY5Y cells (Fig. S4A–D) with α7 nAChR agonist and observed a dose- and time-dependent decrease in the abundance of sirt6, and this reduction was blocked by nAChR antagonists in primary neurons (Fig. S3F) and SH-SY5Y cell cultures (Fig. S4E–F), although d-TC and MLA alone had no effect on sirt6 levels (Fig. S4G–H). Notably, while a clear time-dependent reduction in sirt6 was observed up to 12 h, its level showed a slight rebound at 24 h post-agonist treatment, probably reflecting the transient pharmacokinetics of the agonist or the engagement of endogenous cellular homeostatic mechanisms and holding much implications for the translational relevance. Moreover, in HEK 293 T cells that do not express α7 nAChR, nicotine had no effect on the abundance of sirt6; however, after overexpressing α7 nAChR in HEK 293 T cells, nicotine significantly reduced the level of sirt6 (Fig. S4I–J). Additionally, brain lysate analysis revealed that mice exposed to PNU282987 or nicotine exhibited a time-dependent reduction in sirt6 levels (Fig. S3G). These data collectively consolidated the role of α7 nAChR in downregulation of sirt6 protein level. Furthermore, we investigated whether stimulating α7 nAChR could reduce sirt6 levels after PFF treatment. Western blotting data showed that α-syn PFF treatment significantly increased sirt6 protein levels, and that PNU282987 or nicotine largely abolished this increase in primary neurons (Figs. 2B and S3H) and mouse midbrain (Fig. S3I). Then, we sought to determine whether α7 nAChR-mediated PARP activity inhibition and neuroprotection were dependent on sirt6. Results showed that shRNA-mediated knockdown of sirt6 mimicked the effects of the α7 nAChR agonist, attenuating α-syn PFF-induced PAR accumulation (Figs. 2C and S3M) and cell death (Fig. S3J–L). In contrast, overexpression of sirt6 exacerbated PFF-induced cell death (Fig. S3N–P), enhanced PAR accumulation (Fig. S3Q and S3R), and aggravated α-syn pathology (Fig. S3Q and S3S), suggesting sirt6 is a critical mediator for α7 nAChR to suppress PARP1 activity.

**Figure 2.**
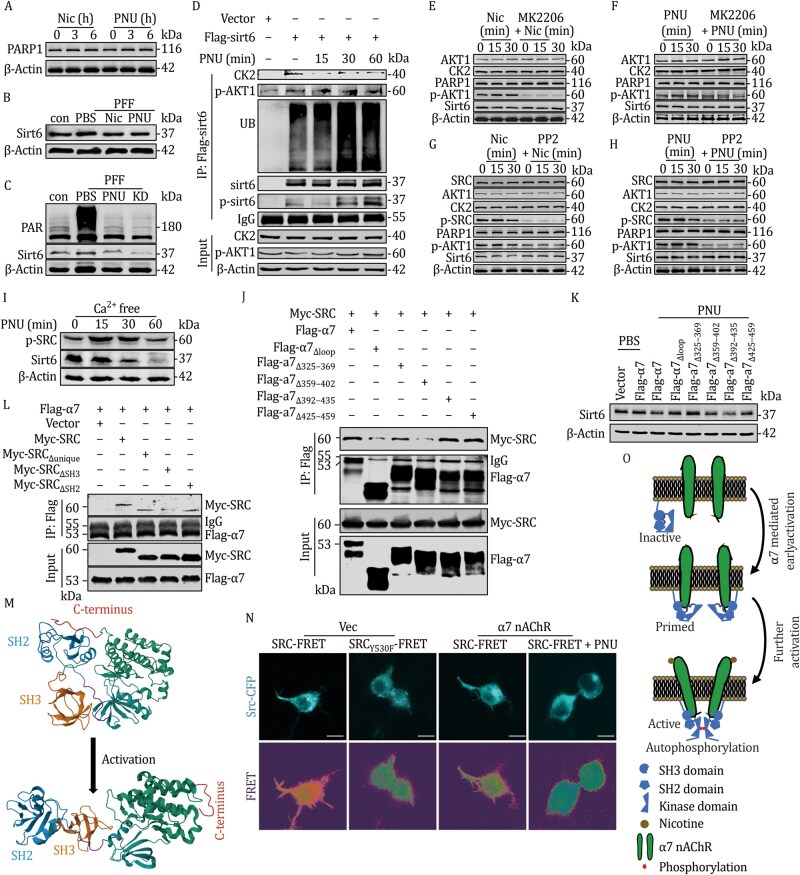
**PARP1 hyperactivation was inhibited by α7 nAChR through a metabolic signalling pathway**. (A) Representative immunoblots of PARP1 levels in primary cortical neurons incubated with nicotine or PNU282987 for 3 or 6 h (*n* = 3). (B) Primary cortical neurons were preincubated with nicotine or PNU282987 for 1 h and further incubated with PFF for 24 h. Sirt6 levels were determined by western blot analysis (*n* = 3). (C) Primary cortical neurons were transduced with lentiviruses containing sirt6 shRNA or pretreated with PNU282987 and further incubated with PFF for 7 days. The PAR level was determined by western blot. (*n* = 3). (D) SH-SY5Y cells were transduced with Flag-sirt6 and then further incubated with MG132 (10 µmol/L) for 2 h, followed by incubation with PNU282987 (10 nmol/L). Cell lysates were subjected to immunoprecipitation using a Flag-tagged antibody, and the levels of ubiquitin, sirt6, CK2, p-AKT1 (S473), and p-sirt6 (S338) were determined by immunoblotting. (E) Primary cortical neurons were preincubated with MK2206 (1 µmol/L) for 1 h and further incubated with nicotine. Cell lysates were harvested for immunoblot analysis (*n* = 3). (F) Primary cortical neurons were treated, and the data were analysed similarly to those in (E) except for incubation with PNU282987 (10 nmol/L). (G) Primary cortical neurons were preincubated with PP2 (10 µmol/L) for 1 h and further incubated with nicotine. Cell lysates were harvested for immunoblot analysis (*n* = 3). (H) Primary cortical neurons were treated, and the data were analysed similarly to those in (G) except for incubation with PNU282987 (10 nmol/L) (*n* = 3). (I) Representative immunoblots of sirt6 and p-SRC in SH-SY5Y cells preincubated with Ca^2+^-free medium for 0.5 h and further incubated with PNU282987 (10 nmol/L) in a time-dependent manner (0 min, 15 min, 30 min, and 60 min) (*n* = 3). (J) Each truncated construct of Flag-α7 was cotransfected with Myc-SRC, NACHO, and RIC3 into HEK 293 T cells. After cultivation for 24 h, the extracted membrane fraction was subjected to immunoprecipitation using an anti-Flag antibody and probed with a Myc-tagged antibody. (K) Representative immunoblots of sirt6 levels in HEK 293 T cells transfected with each truncated construct of Flag-α7, NACHO, and RIC3 for 24 h and further incubated with PNU282987 (10 nmol/L) for 2 h. (L) Each truncated mutant of Myc-SRC was cotransfected with Flag-α7, NACHO, and RIC3 into HEK 293 T cells. After cultivation for 24 h, the lysates were subjected to immunoprecipitation using an anti-Flag antibody and probed with a Myc-tagged antibody. (M) Illustration of the inactive and active SRC structures with critical motifs depicted. Structure of inactive SRC based on Protein Data Bank entry 2SRC; Structure of active SRC based on Protein Data Bank entry 1Y57. (N) HEK293T cells were transiently coexpressing α7 nAChR and SRC-FRET (or SRC-FRETY530F) and further incubated with PNU282987 (10 nmol/L) for 15 min. FRET ratio images of the SRC-FRET biosensor in representative HEK 293 T cells. (O) Schematic illustration of the mechanism by which α7 nAChRs activate SRC.

To determine the mechanism of α7 nAChR-mediated sirt6 downregulation, we first verified whether sirt6 mRNA levels were altered. The qPCR data showed that PNU282987 did not cause any significant alteration in sirt6 mRNA levels (Fig. S5A), implying that sirt6 downregulation might occur at the protein level. Subsequently, it demonstrated that treatment with MG132 (proteasome inhibitor), but not NH_4_Cl (lysosome inhibitor), effectively rescued sirt6 protein levels following α7 nAChR activation (Fig. S5B–C), implying proteasomal degradation pathways might be involved in sirt6 downregulation. To reconsolidate this finding, the sirt6 ubiquitination level was measured. It showed PNU282987 promoted sirt6 ubiquitination and phosphorylation at Ser338 (Fig. 2D), a modification linked to proteasomal degradation (Thirumurthi et al., 2014). Together, these results suggested that stimulation of α7 nAChR could induce sirt6 phosphorylation at Ser338, which in turn led to the degradation of sirt6 through the ubiquitin–proteasome pathway.

Based on the web PhosphoSitePlus, the phosphorylation of sirt6 at Ser338 was mainly conferred by the kinases AKT1 and CK2. Thus, to explore how α7 nAChRs mediate the suppression of sirt6, phosphorylated AKT1 (p-AKT) and CK2 were monitored following the stimulation of α7 nAChRs. It showed that α7 nAChR agonists induced AKT1 phosphorylation (within 15–30 min) and sirt6 degradation, and AKT1 inhibitors (MK2206, capivasertib) abolished these effects in primary neurons (Figs. 2E–F, S5D and S5E) and SH-SY5Y cells (Fig. S6A–E); whereas the levels of CK2 were not affected by α7 nAChR agonists (Figs. 2E–F, S5D and S5E), suggesting phosphorylation and degradation of sirt6 was mediated by AKT1, instead of CK2. By the way, after α7 nAChR agonist treatment for more than 3 h, no significant differences in the p-AKT level were detected (Fig. S5F–G), implying AKT1 phosphorylation occurred rapidly in half of an hour. Furthermore, shRNA-mediated AKT1 downregulation abolished nicotine-induced sirt6 degradation (Fig. S6F); on the contrary, even without α7 nAChR stimulation, the activation of AKT1, by its agonist SC79 (Figs. S5H, S6G and S6H) or transfection of its constitutively active form Myr-AKT (Fig. S6I), was sufficient to decrease the sirt6 level. Collectively, these observations suggested that AKT1 activation is sufficient and necessary for sirt6 degradation upon stimulation of α7 nAChR.

To investigate the mechanism by which α7 nAChR activates AKT1, we identified proteins interacting with α7 nAChR via coimmunoprecipitation followed by LC-MS analysis. Among 143 interactome proteins, GO analysis of the membrane-localized candidates revealed three kinases: PIP5K1A, TGFBR1, and SRC. Since previous studies demonstrated α7 nAChR interacted with the nonreceptor tyrosine kinase SRC, which could phosphorylate and activate AKT1 (Chen et al., 2001), we hypothesize α7 nAChR activates AKT1 through SRC-mediated signaling. Indeed, it showed α7 nAChR agonists induced rapid SRC phosphorylation at Y419 in primary neurons (Figs. 2G, 2H, S7A and S7B) and SH-SY5Y cells (Fig. S8A–D) within half of an hour, since no significant changes in p-SRC levels were observed after α7 nAChR agonist treatment for over 3 h (Fig. S7C–D). Next, we determined whether SRC activation was necessary and sufficient for sirt6 degradation. It showed that the SRC inhibitor PP2 abrogated α7 nAChR-mediated sirt6 degradation and AKT1 activation (Figs. 2G–H, S7A–B). Concomitantly, shRNA-mediated knockdown of SRC attenuated the α7 nAChR-induced reduction in sirt6 levels (Fig. S8E). Moreover, SRC activator YEEI peptide or constitutively active SRC (Y530F) suppressed the sirt6 protein level without α7 nAChR stimulation (Fig. S7E and S6I). Furthermore, we defined the upstream role of SRC in regulating AKT1 activation within this cascade. It showed that sirt6 downregulation induced by the AKT1 agonist SC79 was not affected in the presence of the SRC inhibitor PP2 (Fig. S7F); in contrast, sirt6 downregulation induced by the SRC agonist YEEI was effectively reversed by the AKT1 inhibitor MK2206 (Fig. S7G). Taken together, these data implied that SRC-AKT1 axis might play a role in α7 nAChR suppressing the abundance of sirt6.

Next, we investigated the mechanism through which α7 nAChRs activate SRC. As α7 nAChRs are ligand-gated ion channels that are permeable to Ca^2+^, we first suspected that Ca^2+^ influx through α7 nAChRs might be involved in SRC activation. Surprisingly, chelation of extracellular calcium had little effect on PNU282987-mediated SRC phosphorylation and subsequent sirt6 degradation (Figs. 2I and S9A). Then, considering α7 nAChRs can also play metabolic roles by activating signaling pathways involved in various cellular events (Kabbani and Nichols, 2018), we thus tested the possibility of α7 nAChRs physically priming SRC activation. The chimera analysis revealed that the amino acid residues 325–369 in the intracellular loop of α7 nAChR were essential for its interaction with SRC (Figs. 2J–K and S9B), and truncation of this motif not only abolished their interaction but also inhibited α7 nAChR-mediated sirt6 reduction (Figs. 2J–K and S9B). On the other hand, the immunoprecipitation analysis suggested the SH3 domain of SRC is critical for its binding to α7 nAChR (Fig. 2L). Then, based on the known structures of SRC (Boczek et al., 2019; Cowan-Jacob et al., 2005), we hypothesized that α7 nAChRs physically bind to SRC, leading to destruction of the tight structure of the SRC, which primes SRC activation through α7 nAChR global structural rearrangements during channel opening. Given that SRC activation dramatically increases the distance between the C-terminus and SH2 domain (Boczek et al., 2019; Cowan-Jacob et al., 2005) (Fig. 2M), to dynamically detect this process by FRET (Fig. S9C), SRC-FRET biosensors were constructed (Fig. S9D), then cotransfected with α7 nAChR. The results demonstrated that the FRET ratio was obviously lower compared with group cotransfected with blank vector; notably, PNU282987 treatment further reduced the FRET ratio in cells coexpressing α7 nAChR; not surprisingly, a very low FRET ratio was observed in the SRC_Y530F_-FRET group (Figs. 2N and S9E). These data suggested that upon α7 nAChR binding to SRC, the compact SRC structure became loosened; during the global structural rearrangement of α7 nAChR after agonist stimulation, the bound SRC became more expanded and stretched (Fig. 2O).

This study highlighted that PARP1 activity was suppressed by stimulation of α7 nAChR, which mitigated the α-syn PFF-induced neurotoxicity, and one potential signaling cascade was proposed as illustrated in Fig. S9F, offering novel insights into potential new therapeutic strategies for PD.

## Supplementary Material

pwag004_Supplementary_Data

## Footnotes

All the authors declare that they have no conflict of interest.

C.Z., W.W. and J.Z. designed the entire project and wrote the manuscript; X.R., D.G., and F.X. conducted the experiments; F.Z., J.S., and Y.Z. analysed the data; H.H. and Z.D.X. provided constructive suggestions for this experiment and interpreted the data.

This work was supported by grants from the National Natural Science Foundation of China (32071164) and the Beijing Natural Science Foundation (7242006).

All experiments were authorized by the Institutional Animal Care and Use Committee of Capital Medical University (approval no. AEEI-2016-057) and were performed according to the National Institutes of Health (NIH) *Guide for the Care and Use of Laboratory Animals*.

All the authors declare their agreement to participate.

All the authors declare their agreement to publish.
