# Supplementary material for: PARP1 suppression by α7 nAChR activation attenuated α-synuclein-induced neurotoxicity in Parkinson’s disease
Source: Protein Cell. 2026 Apr 11;17(7):669–74. doi: 10.1093/procel/pwag004 (PMC13340922; doi:10.1093/procel/pwag004)

**MATERIALS AND METHODS**

**Preparation of recombinant α-syn and PFFs**

The α-syn gene was subcloned and inserted into the pGEX-4T-1 vector, expressed in *Escherichia coli* BL21 (DE3) and purified as described previously ([Ren et al., 2019](#_ENREF_1)). After the bacterial endotoxins were removed using a Toxineraser endotoxin removal kit (GeneScript, NJ), the purified α-syn protein was diluted in PBS to 5 mg/ml and agitated in an Eppendorf orbital mixer (1,000 rpm at 37 °C) for 7 days. Then, the protein was centrifuged at 100,000 × g for 30 min at 4 °C to separate the α-syn fibrils (PFFs, in the pellet fraction) from the α-syn monomers (in the supernatant fraction). The α-syn fibrils were washed once with PBS and resuspended in PBS to an equal volume. The supernatant and pellet were resolved by SDS‒PAGE and detected by Coomassie blue staining. Finally, the α-syn PFFs and monomers were aliquoted and stored at -80 °C.

**Cell culture and α-syn PFF treatment**

The human embryonic kidney cell line (HEK 293T) and human neuroblastoma SH-SY5Y cell line were cultured in Dulbecco's modified Eagle's medium (DMEM) supplemented with 10% (v/v) fetal bovine serum (FBS), 50 U/ml penicillin and 50 mg/ml streptomycin. All cells were incubated in a 5% CO_2_ atmosphere at 37 °C.

Primary cortical neurons were prepared from C57BL/6J mouse embryos (days 14-15). All experiments were authorized by the Institutional Animal Care and Use Committee of Capital Medical University (approval no. AEEI-2016-057) and were performed according to the National Institutes of Health (NIH) *Guide for the Care and Use of Laboratory Animals*. Briefly, the dissociated neurons were plated onto poly-L-lysine (Sigma, St. Louis, MO)-coated coverslips or dishes at 20,000–40,000 cells/cm^2^ or 70,000–100,000 cells/cm^2^, respectively. The cells were cultured in neurobasal medium (GIBCO, VA) supplemented with L-glutamine (0.5 mM) and 50×B27 supplement (for a final concentration of 1×; Gibco). Because α-syn is only expressed at mature synapses, neurons were treated with PFF for 7 days *in vitro* (DIV). For PFF treatment, PFF was first diluted in PBS to a final concentration of 0.1 mg/ml and sonicated with 60 pulses. Unless otherwise noted, the neurons were harvested for further analysis at 21 DIV.

**Cell death and viability assessment**

Primary cultured cortical neurons (DIV 7) were treated with 1 μg/ml PFF for 14 days. Hoechst 33342 (7 μM) and propidium iodide (2 μM) staining were used to determine the percentage of cell death. Images were taken by a Zeiss microscope equipped with automated computer-assisted software (Axiovision 4.6, Carl Zeiss, Dublin, CA). Fluorescent images were examined by two independent researchers blinded to experimental conditions; cells exhibiting clear nuclear PI staining will be scored as positive, with discordant counts resolved by a third investigator. Cell viability was determined with the 3-(4,5-dimethylthiazol-2-yl)-2,5-diphenyltetrazolium bromide (MTT; Promega, Madison, WI) assay. MTT was used at a final concentration of 0.5 mg/ml, and the cells were incubated for 4 h. After washing two times with PBS, the formazan crystals were dissolved in 100 μl of DMSO. The absorbance was measured at 490 nm with a microplate reader (PerkinElmer, Waltham, MA).

**Western blotting**

Commonly, tissues or cells were lysed in RIPA buffer (50 mM Tris at pH 7.4, 150 mM NaCl, 1 M EDTA, 0.25% deoxychloric acid, and 1% NP-40) supplemented with protease inhibitor cocktail and phosphatase inhibitor cocktail (Thermo Scientific, Waltham, MA). The cell lysates were centrifuged at 12,000 × g for 40 min at 4 °C, and the supernatant was retained for further analysis. To detect the aggregated form of α-syn, primary neurons or tissues were resuspended in 1% (vol/vol) TX-100/TBS supplemented with protease and phosphatase inhibitors (Thermo Scientific, Waltham, MA), sonicated 10 times with a 3 s pulse and incubated on ice for 30 min. The cell lysates were subsequently centrifuged (100,000 g) at 4 °C for 30 min, after which the supernatant was harvested as the TX-soluble fraction. The pellets were washed with 1% (vol/vol) TX-100/TBS followed by centrifugation at 100,000 g at 4 °C, and the TX-insoluble proteins were extracted with 2% (wt/vol) SDS/TBS and finally reconstituted in an equal volume of SDS/TBS buffer. The protein concentrations of the TX-soluble fraction and the TX-insoluble fraction were determined using a bicinchoninic acid protein assay kit (Thermo Scientific, Waltham, MA). The proteins were resolved by SDS‒PAGE and transferred to NC membranes or PVDF membranes, which were blocked with 5% milk. The membranes were incubated for 1 h at room temperature with the following antibodies: anti-α-syn (BD Biosciences; 1:2000), anti-p-α-syn (S129) (Abcam; 1:1000), anti-sirt6 (CST; 1:1000), anti-p-sirt6 (S338) (Abnova; 1:1000), anti-AKT1 (CST; 1:1000), anti-p-AKT (S473) (CST; 1:1000), anti-SRC (CST; 1:1000), anti-p-SRC (Y416; based on the amino acid numbering of chicken C-Src) (CST; 1:1000), anti-CSNK2A1 (Proteintech; 1:1000), anti-PAR (Enzo; 1:1000), anti-PARP1 (CST; 1:1000), anti-ubiquitin (SANTA CRUZ; 1:500), anti-α7 nAChR (Gene Tex; 1:1000), anti-Myc tag (Abmart; 1:1000), anti-Flag tag (Applygen; 1:1000) or anti-β-actin (GXYbio; 1:5000). The bands were detected with an Odyssey Infrared Imaging System (LI-COR, Lincoln, NE).

**Immunofluorescence staining of cells**

Primary neurons or cultured cells were fixed with 4% (wt/vol) paraformaldehyde and 4% (wt/vol) sucrose in PBS for 15 min. The cells were rinsed five times with PBS and then permeabilized and blocked with 3% (wt/vol) BSA/0.1% (vol/vol) TX-100 for 15 min. Then, the cells were incubated with primary antibody [anti-p-α-syn (Abcam, Cambridge, UK; 1:400)] for 2 h at room temperature. After being rinsed five times with PBS, the cells were incubated with a secondary antibody [rabbit IRDy594 (LI-COR, Lincoln, NE; 1:500)] for 1 h at room temperature. The fluorescence was visualized with a confocal microscope (Leica Microsystems, Tokyo, Japan).

**Coimmunoprecipitation**

The cells were lysed in RIPA buffer (50 mM Tris at pH 7.4, 150 mM NaCl, 1 M EDTA, 0.25% deoxychloric acid, and 1% NP-40) supplemented with protease inhibitor cocktail and phosphatase inhibitor (Thermo Scientific, Waltham, MA), followed by centrifugation at 12,000 × g for 40 min at 4 °C. The supernatant was incubated with an antibody (anti-Flag or anti-Myc antibody) and protein A/G-agarose overnight at 4 °C. After extensive washing with lysis buffer, the bound proteins were eluted from the beads by boiling in loading buffer and subjected to western blotting analyses or liquid chromatography‒mass spectrometry assays.

**Liquid chromatography tandem mass spectrometry**

The eluted protein was digested and processed by FASP (protein digestion by filter-aided sample preparation) with slight modifications. The protein solution was diluted to 500 μL with UA solution (8 M urea in 0.1 M Tris-HCl, pH 8.5). The sample solution was centrifuged on a 30 kDa filter for 20 minutes. Then, 200 μL of UA solution with 10 mM DTT was added, and the reduction reaction was continued for 4 h at 37 °C. The solution was removed by centrifugation, and a UA solution with 50 mM iodoacetamide (IAA) was added. The sample was incubated in the dark for 30 min at room temperature. The ultrafraction tube was washed with 200 μL of UA three times and 200 μL of ABC solution (50 mM ammonium bicarbonate) three times by centrifugation at 14,000 ×g for 15 min at room temperature. Then, 100 μL of ABC containing 0.1 μg/μL of trypsin was added to each ultracentrifugation tube. The tubes were incubated at 37 °C for 12 h. Peptides were collected by centrifugation at 14,000 × g for 15 minutes. The tubes were washed twice with 100 μL of ABC for 15 min while being centrifuged at 14,000 g. The flow fractions were collected together, and the concentration was measured using a NanoDrop 2000C at 280 nm absorbance.

The LC‒MS detection system consisted of a nanoflow HPLC chromatographic instrument (Easy nLC1000 System, Thermo Fisher, USA) coupled to an Orbitrap Fusion mass spectrometer (Thermo Fisher Scientific, USA) with a nanoelectrospray ion source (Thermo Fisher Scientific, USA). Briefly, 0.5 μg of peptide mixture was loaded onto a 2 cm self-packed trap column (100 µm id, ReproSil-Pur C18-AQ, 3 μm; Dr Maisch GmbH, Germany) using flow phase A (99.9% water and 0.1% FA) and separated on a 75 μm inner diameter 12 cm length column (ReproSil-Pur C18-AQ, 3 μm; Dr Maisch GmbH, Germany) using a 78 min linear gradient of 6-32% flow phase B (99.9% acetonitrile and 0.1% FA) at a flow rate of 300 nl/min.

**Real-time reverse polymerase chain reaction (RT‒PCR)**

Total RNA was extracted from cultured cells using an RNAsimple Total RNA Kit (TIANGEN, Beijing). A cDNA library was prepared using a Superscript III Synthesis System (Vazyme, Nanjing). RT‒PCR was performed using a ChamQ Universal SYBR qPCR Master Mix kit (Vazyme, Nanjing), and the results were detected with an iCycler Real-Time PCR Detection System (Bio-Rad, Hercules). GAPDH was used as an internal control. The sequences of primers used in this analysis were as follows: sirt6-F: TGTGGAAGAATGTGCCAAGTGT; sirt6-R: AGCGATGTACCCAGCGTGAT; GAPDH-F: AAAGGGTCATCATCTCTG; and GAPDH-R: GCTGTTGTCATACTTCTC.

**FRET analysis**

To generate SRC-FRET biosensor constructs, the full-length human SRC gene was subcloned and inserted into the pCDNA3.1(+) vector. The cDNA of the donor fluorophore mECFP was inserted into the SH2 domain of SRC (between amino acids 211 and 212 of SRC). To minimize the potential disruptive effect of fluorescent protein insertion on SH2 domain folding, two highly flexible linker peptides (DVGRAEVVV and DVTSTAATTS) were added to the donor fluorescent protein inserted into the SH2 domain. The acceptor fluorescence protein mCitrine was linked to the C-terminus of SRC with a flexible linker (EFGGSGG). As a positive control, mECFP and mCitrine were directly linked via a flexible linker (EFGGSGG).

FRET analysis by live-cell imaging was performed using confocal microscopy of HEK 293T cells. Confocal microscopy was performed 48 hours after plasmid transfection. Using filter settings for the donor channel (λex458 nm, λem462-510 nm), acceptor channel (λex514 nm, λem518-580 nm) and FRET channel (λex458 nm, λem518-580), three images were captured, and FRET was subsequently calculated automatically. All confocal images were acquired using a Leica Microsystems confocal microscope (Leica Microsystems, Tokyo, Japan). During the measurements, regions with intensities that were too high were excluded. All settings for detector gain and laser power were selected at the beginning of imaging and not altered during FRET measurements. Background correction was conducted by defining and averaging a region of interest (ROI) outside of the cell and excluding all pixels below this value plus 3-fold its standard deviation. Areas that showed overexpression were excluded as well. Cross-talk factors were calculated by setting and averaging an ROI within cells, which expressed donor and acceptor only. FRET efficiencies were then calculated pixelwise in a batch procedure for all images, and FRET efficiency images of the cells were graphed.

**Lentiviral construction and infection**

The shRNA sequences targeting specific genes were subcloned and inserted into pLVX-shRNA2 lentiviral vectors, and green fluorescent protein (ZsGreen) was used as an indicator of transfection efficiency. The following target sequences were used: mouse α7 nAChR, 5’-GATGAGAGTGAGGTGATCT3’, and its scrambled control, 5’-GG TTATAGCGAGGTTAGGA3’; human AKT1, 5’-TGACCATGAACGAGTTTGA3’, and its scrambled control, 5’-GAGTCCCTAAAGTGAGTAT3’; mouse sirt6, 5’-GCAGTGCATGTT TCGTATA3’, and its scrambled control, 5’- GATCGGTTAGTACTTCAGT3’; and human SRC, 5’-GTACCTGCGGCTGCCTCAG3’, its scrambled control, 5’-GCCTGGCTCGTGGCTACCA3’. For common knockdown experiments, shRNA-encoding plasmids were transfected into SH-SY5Y cells with PEI, and 48 hours after transfection, subsequent analyses were performed. To produce lentiviruses, the shRNA-encoding lentiviral plasmid was cotransfected with lentivirus package helper plasmids (pMD2. G and pSPAX2) into HEK 293T cells. Lentiviral supernatants were collected, filtered through 0.45-μm filters, and used to transduce primary neuronal cells. Primary cortical neurons were cultured in 6-well plates, and after 5 days of cultivation, lentiviral particles were added to the wells at a multiplicity of infection (MOI) of 5 and incubated overnight.

**Stereotaxic injections and α7 nAChR agonist treatment**

C57BL/6J mice between 2 and 3 months of age were deeply anesthetized with a mixture of ketamine (100 mg/kg; Bela-Pharm) and xylazine (10 mg/kg; Bela-Pharm) and then stereotaxically injected with α-syn PFF (5 μg) by inserting a single needle into the right dorsal neostriatum (coordinates: +0.2 mm relative to bregma, +2.0 mm from midline, +2.6 mm beneath the dura). Injections were performed at a rate of 0.2 μl/min (2.5 μl total per site) with the needle in place for > 5 min at each target. The animals were monitored regularly following recovery from surgery.

For α7 nAChR agonist treatment, the agonists were administered to the mice 1 day before α-syn PFF injection. For nicotine, -(-) nicotine tartrate was dissolved in the drinking water of the mice at a concentration of 200 μg/ml, and the nicotine solutions were freshly added 3 times a week. For PNU282987, the mice received intraperitoneal injections of PNU282987 at a dose of 1 mg/kg/3 days. To exclude possible effects of agonist administration on mouse behavior, α7 nAChR agonist-treated mice were suspended for one week before the behavioral experiments.

**Behavioral experiments**

Behavioral experiments were performed 30, 90 or 180 days after PFF injection. The mice were habituated to the testing room 1 h before the tests, and the apparatuses were cleaned with 70% ethanol between the animals to minimize odor cues. For the rotarod test, each mouse was first pretrained (four 5-min trials, 5 min apart) to acclimate to the rotarod apparatus. During the test period, each mouse was placed on the rotarod at increasing speeds, from 4 to 40 rpm, for 300 s. The latency to fall off the rotarod within this time period was recorded. Each mouse underwent two consecutive trials, and the mean latency to fall in each group was used in the analysis. The wire hang test was carried out as previously described ([Ren et al., 2019](#_ENREF_1)). The mice were placed on the top of a standard wire cage lid, which was lightly shaken to cause the animals to grip the wires and then turned upside down. The latency of the mice to fall off the wire grid was recorded, and average values were computed from two trials (15 min apart). Trials were stopped if the mouse remained on the lid after 5 min. We used 12 female mice for behavioral analysis for each group, and all tests were conducted by an experimenter blinded to the treatment group.

**Immunofluorescence and immunohistochemistry of brain slices**

Mice were anesthetized and perfused with physiological saline followed by 4% paraformaldehyde/PBS, and the brains were removed, fixed in 4% paraformaldehyde overnight and transferred to 30% sucrose for cryoprotection. Then, the brains were sectioned at a thickness of 40 μm, and the sections were incubated in 0.3% TX-100/PBS for 60 min. After washing with PBS, the sections were subjected to antigen retrieval by heating at 95 °C for 10 min in sodium citrate buffer.

For immunofluorescence experiments, sections were incubated in 10% goat serum in 0.1% Triton X-100/PBS for 60 min and then incubated in anti-p-α-syn (1:400; Abcam, Cambridge, UK) for 2 h at room temperature. After washing with PBS, the brain slices were incubated with the secondary antibody rabbit IRDy594 (1:500; LI-COR, Lincoln, NE) for 1 h at room temperature. The fluorescence was visualized and analyzed with a confocal microscope (Leica Microsystems, Tokyo, Japan).

For immunohistochemistry experiments, the sections were incubated in 0.3% TX-100/PBS for 60 min and then in 3% H_2_O_2_ for 10 min to block endogenous peroxidase activity. After washing in PBS, the sections were incubated in 10% goat serum in 0.1% Triton X-100/PBS for 60 min and then incubated with anti-TH (1:8000; Sigma, St. Louis, MO) for 2 h at room temperature, followed by incubation with biotinylated rabbit anti-rat immunoglobulin G (IgG, 1:200; Vector Laboratories, Burlingame, CA) for 1 h at room temperature. After washing two times, ABC reagent (Vector Laboratories, Burlingame, CA) was added, and the sections were developed using DAB peroxidase substrate. Permanently mounted slides were observed and photographed using a microscope equipped with a digital imaging system (DSRi2, Nikon). For quantification, one of every six substantia nigra serial sections was selected for TH^+^ neuron counting. Cells with a certain area of the fluorescent signal (serially detected with more than 25 pixels in the binary black‒white version of the image) were judged to be TH positive and were counted by ImageJ software.

**Statistical analysis**

The statistical analysis was conducted using GraphPad Prism software (version 6, GraphPad, La Jolla, CA). The data are presented as the mean ± SEMs of at least three independent experiments. One-way ANOVA followed by Tukey’s post hoc test was used for statistical analysis. n.s., no significant difference. A p value of 0.05 or lower was considered to indicate statistical significance.

**References**

Ren, X., Zhao, Y., Xue, F., Zheng, Y., Huang, H., Wang, W., Chang, Y., Yang, H., and Zhang, J. (2019). Exosomal DNA Aptamer Targeting alpha-Synuclein Aggregates Reduced Neuropathological Deficits in a Mouse Parkinson's Disease Model. Mol Ther Nucleic Acids 17, 726-740.

**Supplementary figure legends**

**Figure S1.** **PFF-induced PARP1 hyperactivation and related pathology were inhibited by stimulation of α7 nAChR in neurons.** (A) Quantification of PAR levels in Figure 1A (n = 3). (B) Representative images of Hoechst and PI staining of primary cortical neurons preincubated with nicotine or/and ABT888 (1 μM) and further incubated with PFF for 14 days. Scale bar, 20 μm. (C) Quantification for the percentage of PI-positive cells (n = 3). (D) MTT cell viability assay of primary cortical neurons preincubated with nicotine in a concentration-dependent manner (1 μM, 10 μM and 100 μM) in response to PFF (n = 6). (E) Representative images of Hoechst and PI staining of primary cortical neurons preincubated with nicotine, d-TC (10 μM) + nicotine, MLA (10 nM) + nicotine, or PNU282987 (10 nM) for 1 hour and further incubated with PFF for 14 days. Scale bar, 20 μm. (F) Quantification for the percentage of PI-positive cells (n = 3). (G) MTT cell viability assay of primary cortical neurons preincubated with nicotine (100 μM), d-TC (10 μM) + nicotine, MLA (10 nM) + nicotine or PNU282987 (10 nM) in response to PFF (n = 6). (H) Primary cortical neurons were preincubated with PNU282987 for 1 hour and further incubated with PFF for 7 days (n = 3). The PAR level was determined by western blot. (I-J) Quantification of p-α-syn in primary cortical neurons treated with α-syn PFF, α-syn PFF + nicotine or α-syn PFF + PNU282987 for 7 (I) or 14 days (J), as shown in Figure 1C (n = 3). (K-L) Quantification of α-syn (K) and p-α-syn (L) in insoluble fractions from primary cortical neurons treated with PFF, PFF + nicotine or PFF + PNU282987 for 7 or 14 days, as shown in Figure 1D (n = 3). (M) Quantification of the percentage of PI-positive cells in Figure 1E (n = 3). (N) MTT cell viability assay of primary cortical neurons transduced with lentiviruses containing scramble shRNA control or α7 nAChR shRNA and further incubated with PFF for 14 days (n = 6). (O) Primary cortical neurons were transduced with lentiviruses containing scramble shRNA control or α7 nAChR shRNA and further incubated with PFF for 7 days. Lysates were subjected to immunoblotting using antibodies against PAR, α7 nAChR, p-α-syn and β-actin (n = 3). The values are presented as the means ± SEMs. One-way ANOVA followed by Tukey’s post hoc test was used for statistical analysis. n.s., no significant difference; UD undetectable. P < 0.05 (*), P < 0.01 (**), P < 0.001 (***), P < 0.0001 (****).

**Figure S2. PFF-induced PARP1 hyperactivation and related pathology were prevented by activation of α7 nAChR *in vivo***. (A) Quantification of PAR levels in the striatum of mice at 3 months after α7 nAChR agonist exposure or striatal PFF injection, as shown in Figure 1F (n = 3). (B) Quantification of α-syn and p-α-syn levels in Figure 1G (n = 3). (C) Bar graph quantitation of the p-α-syn level in the cortex of mice at 90 days and 180 days, as shown in Figure 1H (n = 3). (D) Stereological SNpc DA neuron counts in Figure 1I (n = 3). (E) Density of striatal dopaminergic terminals in Figure 1I (n = 3). (F-G) Behavioral abnormalities of PBS-, PFF-, PFF + nicotine- or PFF + PNU282987-treated mice at 30, 90, and 180 days were measured by the wire hang test (F) and the grip rotarod test (G) (n = 22 mice for 30 days, 16 mice for 90 days and 6-7 mice for 180 days). (H) Primary neurons were preincubated with nicotine or/and ABT888 (1 μM) for 1 h and further treated with MNNG (50 μM) for 15 min followed by additional 4 h culture. Lysates were subjected to immunoblotting using antibodies against PAR and β-actin (n = 3). (I) The effect of PNU282987 on regulating PARP1 activity was further assessed using a protocol similar to that in (H) (n = 3). The values are presented as the means ± SEMs. One-way ANOVA followed by Tukey’s post hoc test was used for statistical analysis. UD undetectable. P < 0.05 (*), P < 0.01 (**), P < 0.001 (***), P < 0.0001 (****).

**Figure S3. PARP1 hyperactivation was suppressed via α7 nAChR activation-mediated reduction of sirt6 protein levels.** (A) Representative immunoblots and quantification of PARP1 levels in primary cortical neurons incubated with gradient concentrations of nicotine or PNU282987 for 24 hours (n = 3). (B) Quantification of PARP1 levels in primary cortical neurons incubated with nicotine or PNU282987 for 3 or 6 hours, as shown in Figure 2A (n = 3). (C) Western blot analysis of sirt6 in primary cortical neurons incubated with nicotine or PNU282987 in a concentration-dependent manner (n = 3). (D-E) Western blot analysis of sirt6 in primary cortical neurons incubated with nicotine (D) or PNU282987 (E) in a time-dependent manner (n = 3). (F) Primary cortical neurons were preincubated with d-TC or MLA for 1 hour and further incubated with nicotine for 6 hours. Sirt6 levels were determined by western blot (n = 3). (G) Representative immunoblots and quantification of sirt6 levels in the midbrains of nicotine- or PNU282987-treated mice in a time-dependent manner (n = 3). (H) Quantification of sirt6 levels in Figure 2B (n = 3). (I) Representative immunoblots and quantification of sirt6 levels in midbrain lysates from PBS-, PFF-, PFF + nicotine- or PNU282987-treated mice (n = 3). (J) Representative images of Hoechst and PI staining of primary cortical neurons transduced with lentiviruses containing sirt6 shRNA and further incubated with α-syn PFF for 14 days. Scale bar, 20 μm. (K) Quantification of the percentage of PI-positive cells (n = 3). (L) MTT cell viability assay (n = 6). (M) Quantification of PAR levels in Figure 2C (n = 3). (N-S) Primary neurons were transduced with lentivirus to overexpress SIRT6, followed by treatment with PFF for 7 days. (N) Representative images of Hoechst and PI staining. (O) Quantification for the percentage of PI-positive cells (n = 3). (P) MTT cell viability assay (n = 3). (Q-S) Representative immunoblots and quantification of PAR and p-α-syn levels (n = 3). The values are presented as the means ± SEMs. One-way ANOVA followed by Tukey’s post hoc test was used for statistical analysis. n.s., no significant difference. P < 0.05 (*), P < 0.01 (**), P < 0.001 (***), P < 0.0001 (****).

**Figure S4. Nicotine decreased sirt6 protein levels through α7 nAChRs in cultured cells.** (A) Representative immunoblots and quantification of sirt6 levels in SH-SY5Y cells incubated with nicotine in a concentration-dependent manner for 2 h (n = 3). (B) Representative immunoblots and quantification of sirt6 levels in SH-SY5Y cells incubated with nicotine (100 μM) in a time-dependent manner (n = 3). (C) Representative immunoblots and quantification of sirt6 levels in SH-SY5Y cells incubated with PNU282987 in a concentration-dependent manner for 2 h (n = 3). (D) Representative immunoblots and quantification of sirt6 levels in SH-SY5Y cells incubated with PNU282987 (10 nM) in a time-dependent manner (n = 3). (E) Representative immunoblots and quantification of sirt6 levels in SH-SY5Y cells preincubated with various concentrations of d-TC for 1 h and further incubated with nicotine (100 μM) for 2 h (n = 3). (F) Representative immunoblots and quantification of sirt6 levels in SH-SY5Y cells preincubated with MLA in a concentration-dependent manner for 1 h and further incubated with nicotine (100 μM) for 2 h (n = 3). (G) Representative immunoblots and quantification of sirt6 levels in SH-SY5Y cells incubated with d-TC in a concentration-dependent manner for 1 hour (n = 3). (H) Representative immunoblots and quantification of sirt6 levels in SH-SY5Y cells incubated with MLA in a concentration-dependent manner for 1 hour (n = 3). (I) α7 nAChR was cotransfected with helper (RIC3 and NACHO) into HEK 293T cells, which were then cultivated for 24 h and further incubated with nicotine in a concentration-dependent manner for 2 h (n = 3). (J) Representative immunoblot of α7 nAChR in whole-cell lysates from HEK293T and SH-SY5Y cells (n = 3). The values are presented as the means ± SEMs. One-way ANOVA followed by Tukey’s post hoc test was used for statistical analysis. n.s., no significant difference. P < 0.05 (*), P < 0.01 (**), P < 0.001 (***), P < 0.0001 (****).

**Figure S5. Stimulation of α7 nAChR activated AKT1 and promoted sirt6 degradation through the proteasome pathway.** (A) SH-SY5Y cells were treated with PNU282987 (10 nM) for 1, 3, 6 or 24 hours, RNA was isolated, and qRT‒PCR was performed for sirt6 (n = 3). (B) SH-SY5Y cells were preincubated with NH_4_Cl in a concentration-dependent manner for 4 hours and further incubated with PNU282987 for 2 hours. Sirt6 levels were determined by western blot analysis (n = 3). (C) SH-SY5Y cells were preincubated with MG132 in a concentration-dependent manner for 2 hours and further incubated with PNU282987 for 2 hours. Sirt6 levels were determined by western blot analysis (n = 3). (D) Quantification of sirt6, p-AKT1 and CK2 levels in Figure 2E (n = 3). (E) Quantification of sirt6, p-AKT1 and CK2 levels in Figure 2F (n = 3). (F) Primary neurons were preincubated with MK2206 (1 μM) for 1 h and further incubated with nicotine (100 μM) for 0 h, 3 h and 6 h. Lysates were subjected to immunoblotting using antibodies against AKT1, CK2, PARP1, sirt6, β-actin and p-AKT1 (S473) (n = 3). (G) Primary neurons were treated, and the data were analyzed similarly to those in (F) except for incubation with PNU282987 (10 nM) (n = 3). (H) Representative immunoblots and quantification of AKT1, CK2, PARP1, sirt6, β-actin and p-AKT1 in primary cortical neurons incubated with SC79 (4 μg/mL) in a time-dependent manner (n = 3). The values are presented as the means ± SEMs. One-way ANOVA followed by Tukey’s post hoc test was used for statistical analysis. n.s., no significant difference. P < 0.05 (*), P < 0.01 (**), P < 0.001 (***), P < 0.0001 (****).

**Figure S6. AKT1 activation is sufficient and necessary for sirt6 degradation in SH-SY5Y cells.** (A) Representative immunoblots and quantification of p-AKT1 (S473) in SH-SY5Y cells treated with nicotine (100 μM) for 0 min, 15 min, 30 min and 60 min (n = 3). (B) Representative immunoblots and quantification of p-AKT1 (S473) in SH-SY5Y cells treated with 0-10^3^ μM nicotine for 1 h (n = 3). (C) Representative immunoblots and quantification of p-AKT1 (S473) in SH-SY5Y cells treated with 10 nM PNU282987 in a time-dependent manner (15, 30 and 60 min) (n = 3). (D) Representative immunoblots and quantification of p-AKT1 (S473) in SH-SY5Y cells treated with 0-10^3^ nM PNU282987 for 1 h (n = 3). (E) SH-SY5Y cells were preincubated with the AKT1 blocker capivasertib in a concentration-dependent manner (0.1 μM, 1 μM and 10 μM) for 30 min and then treated with nicotine for 2 h. Sirt6 levels were determined by western blot analysis (n = 3). (F) SH-SY5Y cells were transduced with AKT1 shRNA for 48 h and further incubated with nicotine (100 μM) for 1 h or 2 h. Sirt6 levels were determined by western blot analysis (n = 3). (G) SH-SY5Y cells were incubated with the AKT1 agonist SC79 in a concentration-dependent manner for 2 h. Sirt6 levels were determined by western blot analysis (n = 3). (H) SH-SY5Y cells were incubated with the AKT1 agonist SC79 (4 μg/mL) in a time-dependent manner. Sirt6 levels were determined by western blot analysis (n = 3). (I) Representative immunoblots and quantification of sirt6 levels in SH-SY5Y cells transfected with a constitutively active AKT1 mutant (Myr-AKT1) or a constitutively active SRC mutant (Y530F) for 12 h or 24 h. Sirt6 levels were determined by western blot analysis (n = 3). The values are presented as the means ± SEMs. One-way ANOVA followed by Tukey’s post hoc test was used for statistical analysis. n.s., no significant difference. P < 0.05 (*), P < 0.01 (**), P < 0.001 (***), P < 0.0001 (****).

**Figure S7. α7-nAChR mediates AKT1 activation by activating SRC.** (A) Quantification of sirt6, p-SRC, p-AKT1 and CK2 levels in Figure 2G (n = 3). (B) Quantification of sirt6, p-SRC, p-AKT1 and CK2 levels in Figure 2H (n = 3). (C) Primary neurons were preincubated with PP2 (10 μM) for 1 h and further incubated with nicotine (100 μM) for 0 h, 3 h and 6 h. Lysates were subjected to immunoblotting using antibodies against SRC, AKT1, CK2, PARP1, sirt6, β-actin, p-AKT1 (S473) and p-SRC (Y416) (n = 3). (D) Primary neurons were treated, and the data were analyzed similarly to those in (C) except for incubation with PNU282987 (10 nM) (n = 3). (E) Primary cortical neurons were incubated with the SRC activator YEEI (1 μM) in a time-dependent manner, and then, the sirt6 protein was immunoblotted and analyzed (n = 3). (F) Primary cortical neurons were preincubated with PP2 (10 μM) for 1 h and further incubated with SC79 (4 μg/mL), after which the sirt6 protein was immunoblotted and analyzed (n = 3). (G) Primary cortical neurons were preincubated with MK2206 (1 μM) for 1 h and further incubated with YEEI (1 μM), after which the sirt6 protein was immunoblotted and analyzed (n = 3). The values are presented as the means ± SEMs. One-way ANOVA followed by Tukey’s post hoc test was used for statistical analysis. n.s., no significant difference. P < 0.05 (*), P < 0.01 (**), P < 0.001 (***), P < 0.0001 (****).

**Figure S8. Effects of the α7 nAChR agonists on p-SRC in SH-SY5Y cells.** (A) Representative immunoblots and quantification of p-SRC (Y416) in SH-SY5Y cells treated with nicotine (100 μM) in a time-dependent manner (n = 3). (B) Representative immunoblots and quantification of p-SRC (Y416) in SH-SY5Y cells incubated with nicotine in a dose-dependent manner for 1 h (n = 3). (C) Representative immunoblots and quantification of p-SRC (Y416) in SH-SY5Y cells treated with PNU282987 (10 nM) in a time-dependent manner (n = 3). (D) Representative immunoblots and quantification of p-SRC (Y416) in SH-SY5Y cells treated with PNU282987 in a dose-dependent manner for 1 h (n = 3). (E) SH-SY5Y cells were transduced with SRC shRNA for 48 h and further incubated with nicotine (100 μM) for 1 h or 2 h. Sirt6 levels were determined by western blot analysis (n = 3). The values are presented as the means ± SEMs. One-way ANOVA followed by Tukey’s post hoc test was used for statistical analysis. n.s., no significant difference. P < 0.05 (*), P < 0.01 (**), P < 0.001 (***), P < 0.0001 (****).

**Figure S9.** **α7 nAChR primed SRC activation through global structural rearrangements.** (A) Quantification of sirt6 and p-SRC levels in SH-SY5Y cells preincubated with Ca^2+^-free medium for 0.5 h and further incubated with PNU282987 (10 nM) in a time-dependent manner (0 min, 15 min, 30 min and 60 min) (n = 3). (B) Quantification of sirt6 levels in HEK 293T cells transfected with each truncated construct of Flag-α7, Myc-SRC, NACHO and RIC3 for 24 h and further incubated with PNU282987 (10 nM) for 2 h (n = 3). (C) The proposed model of SRC biosensor action. (D) Schematic representation of the different SRC chimeras used in this study. UD, unique domain; CFP, mECFP; YFP, mCit. (E) The bar graph represents the ratio of FRET, as shown in Figure 2N (n = 11-16 per group). (F) Mechanistic scheme proposing a signaling pathway through which α7 nAChR activation suppresses PFF-mediated neurotoxicity. The values are presented as the means ± SEMs. One-way ANOVA followed by Tukey’s post hoc test was used for statistical analysis. P < 0.05 (*), P < 0.01 (**), P < 0.001 (***).

**Supplementary Figures**


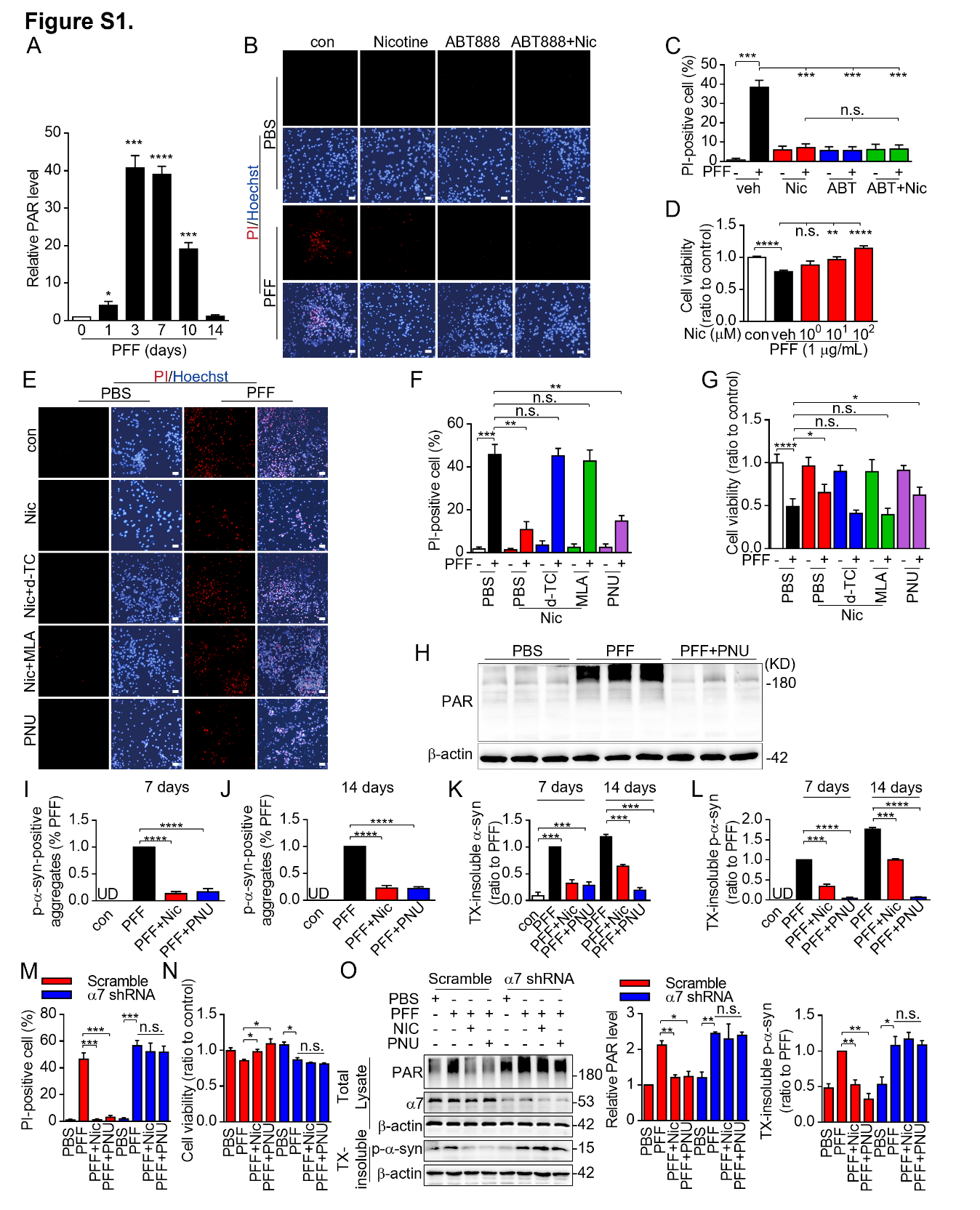


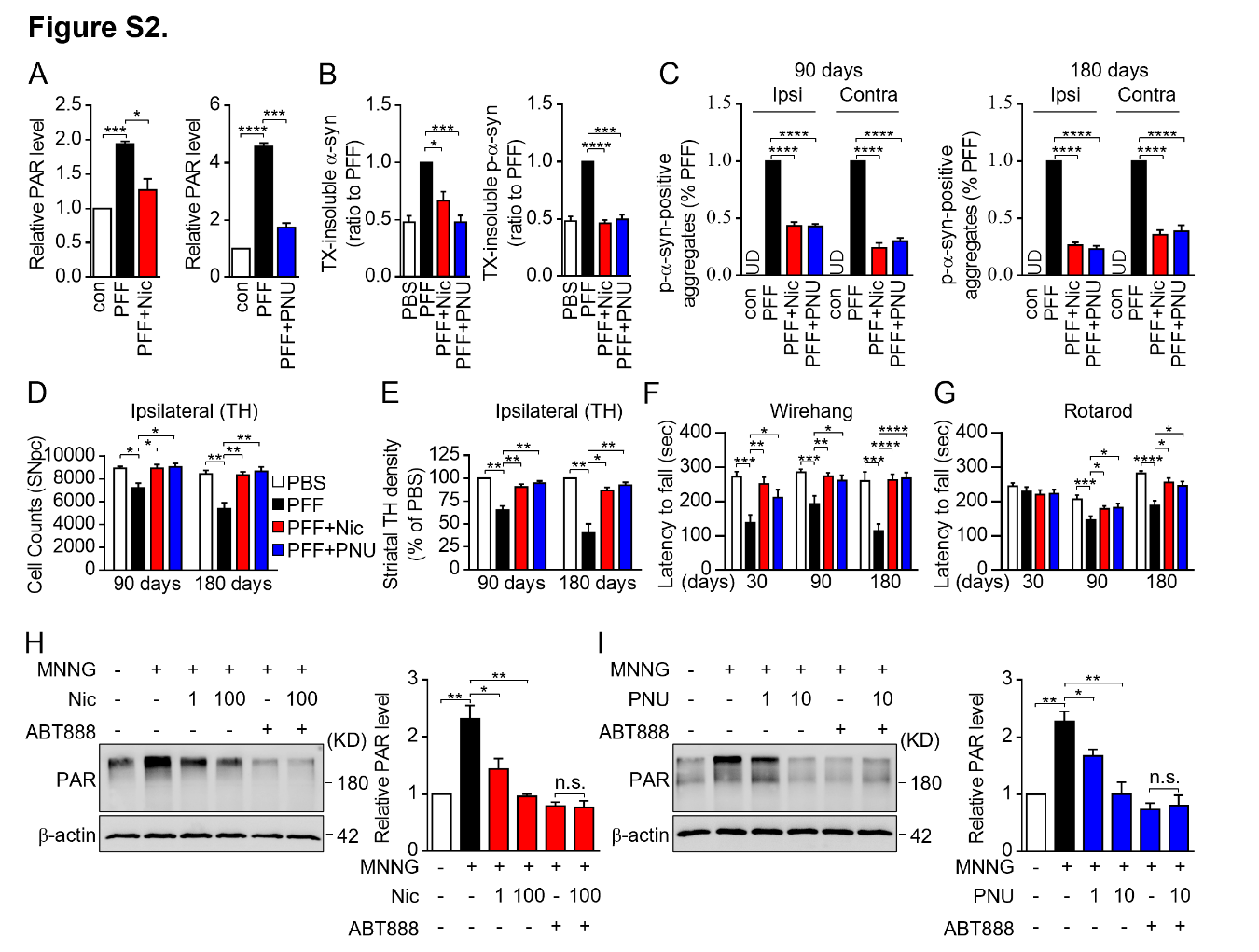


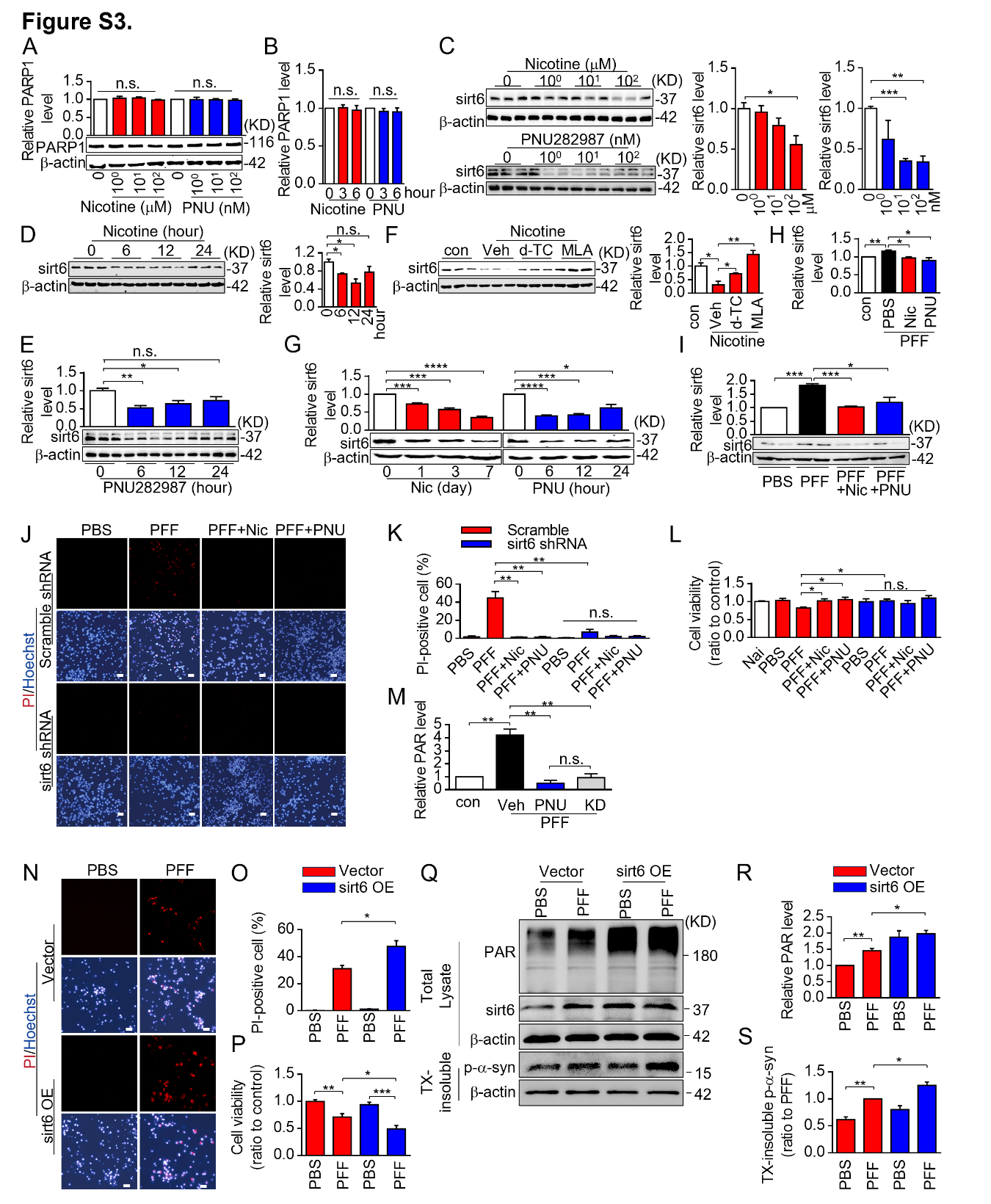


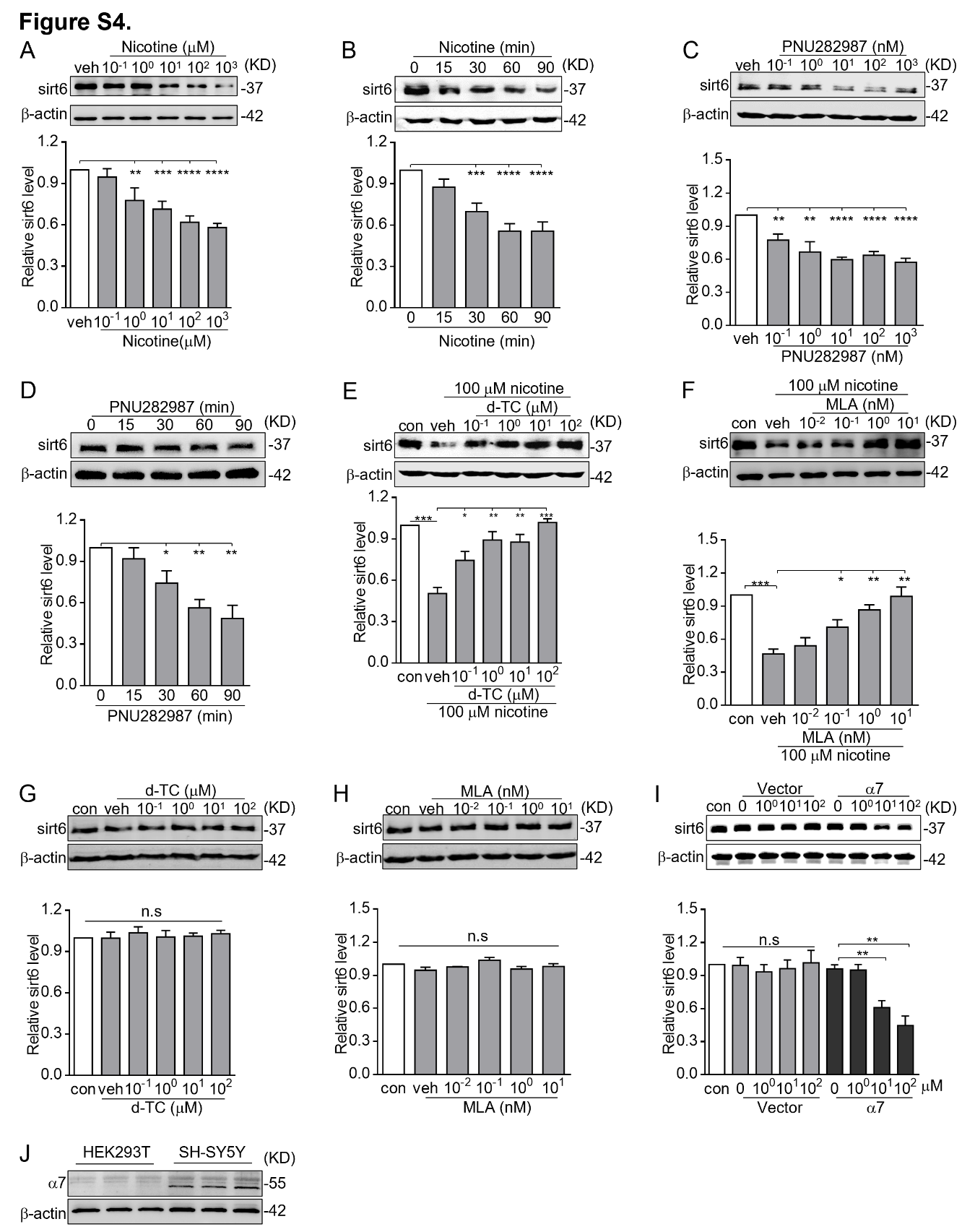


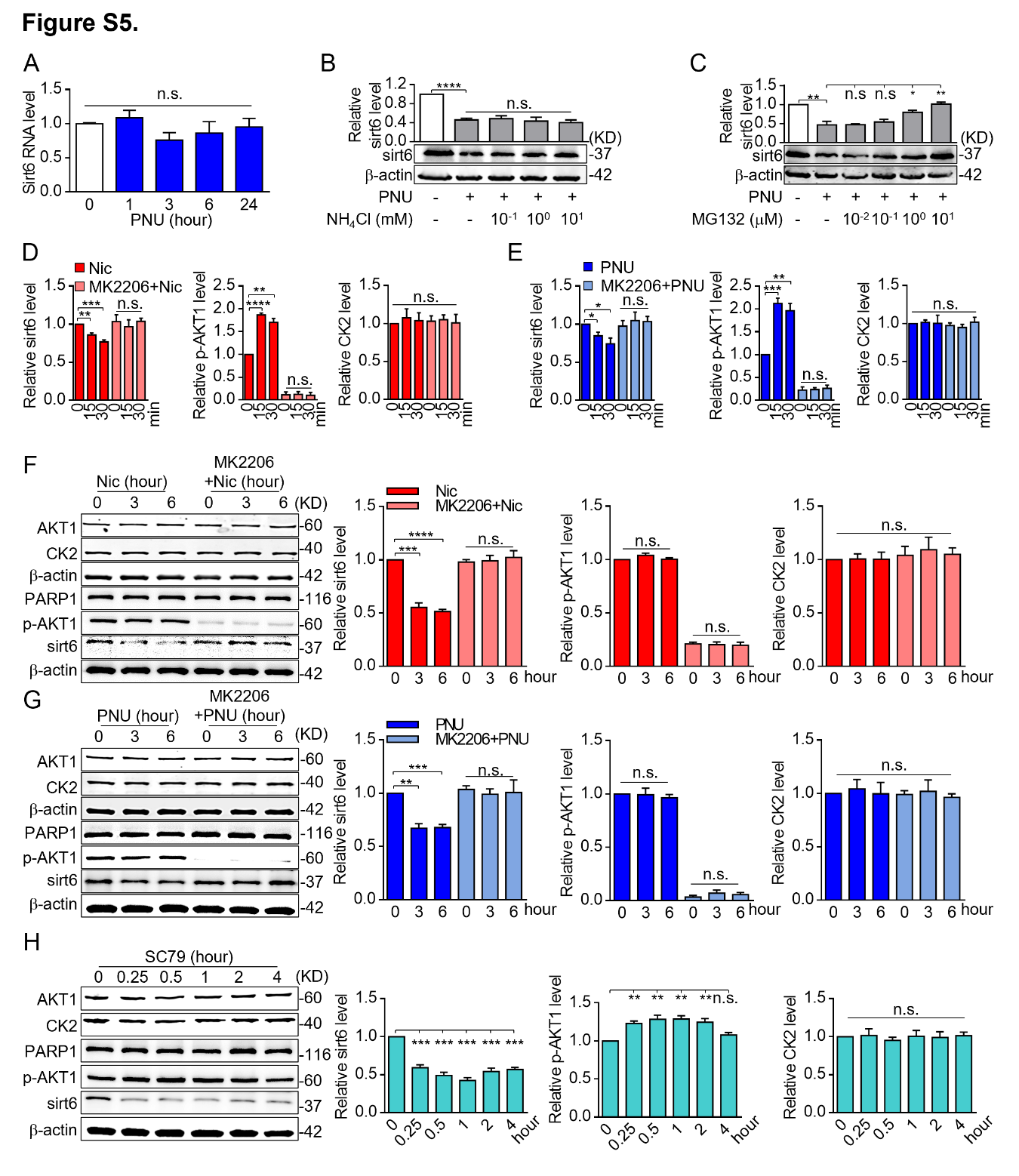


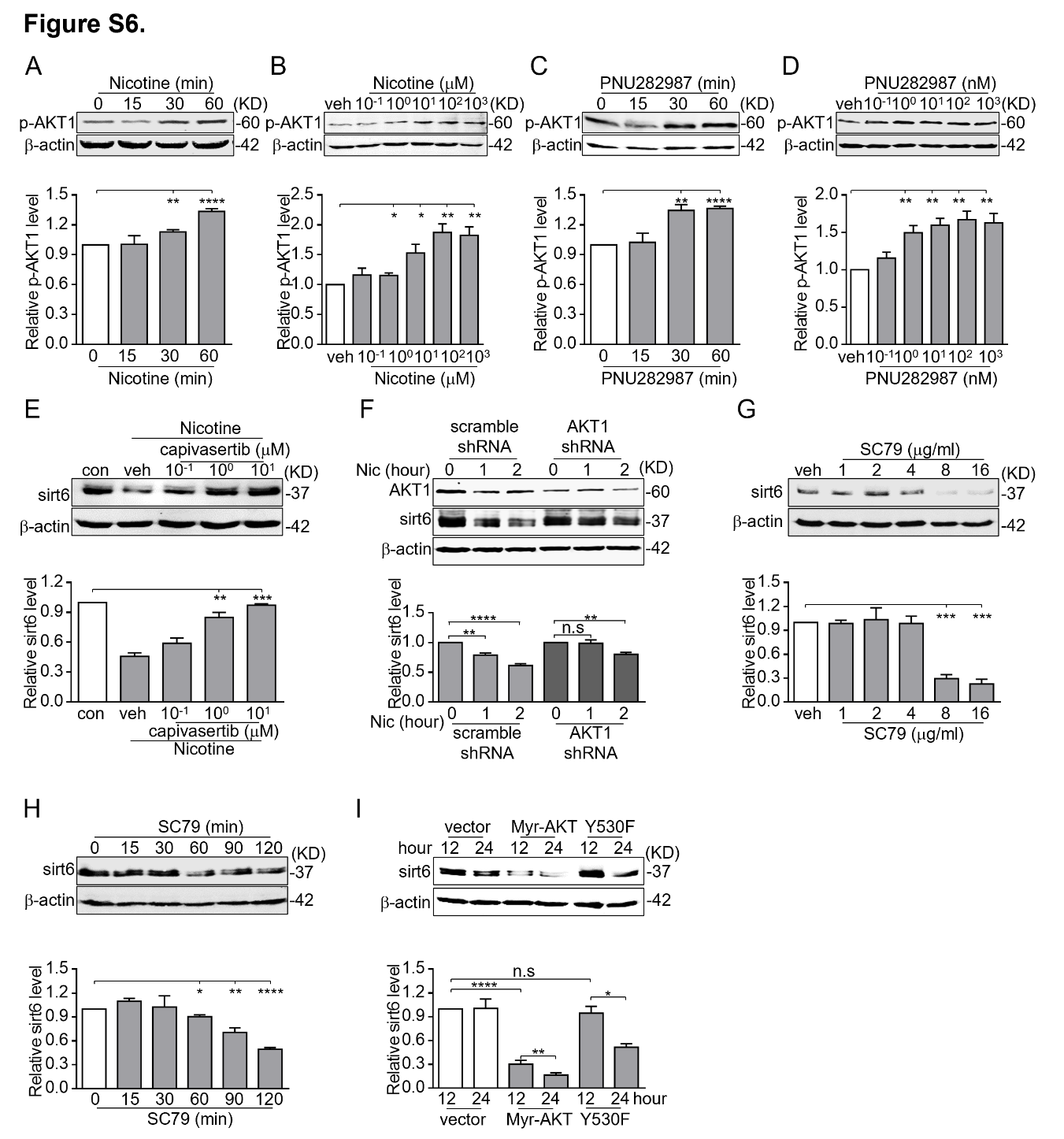


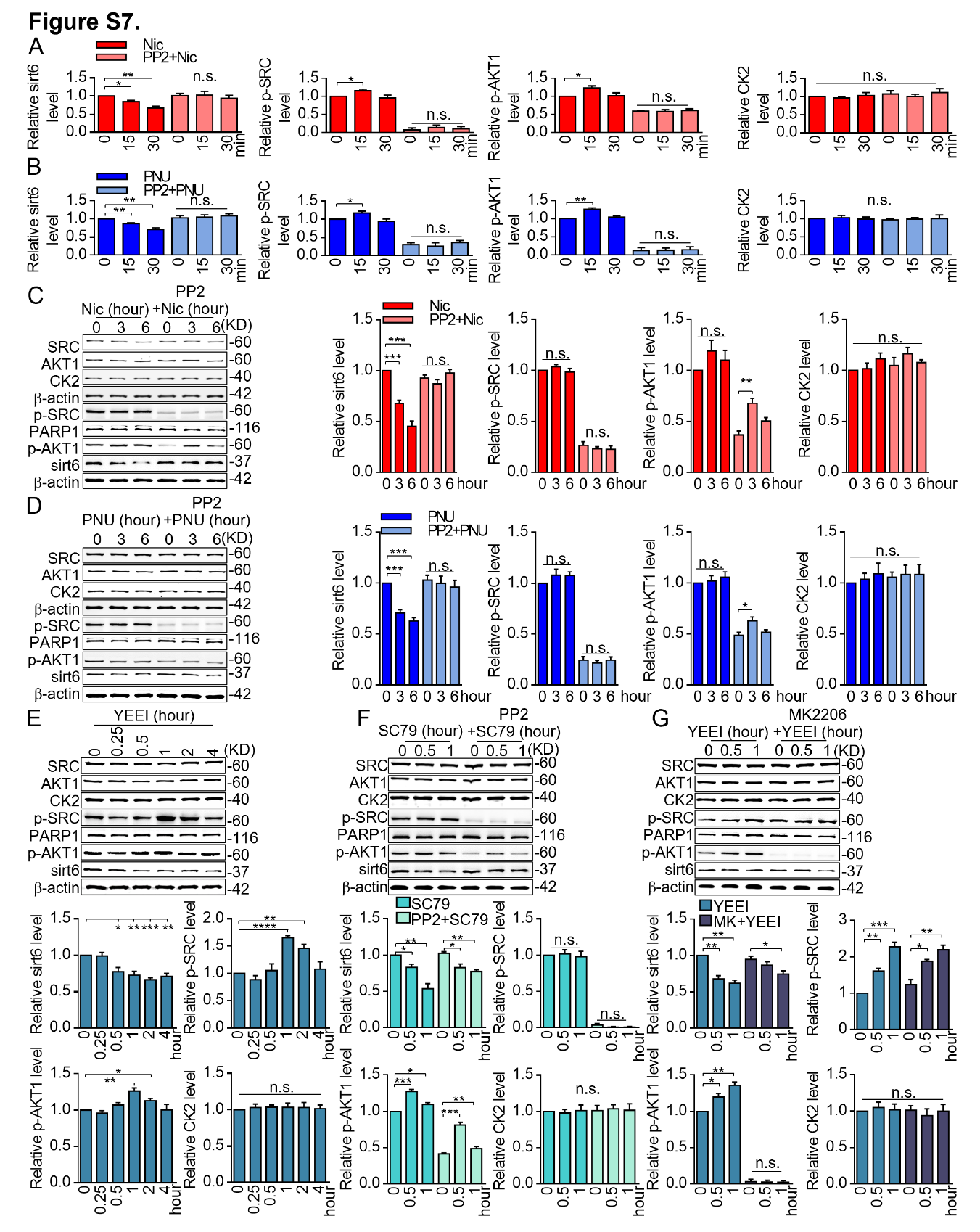


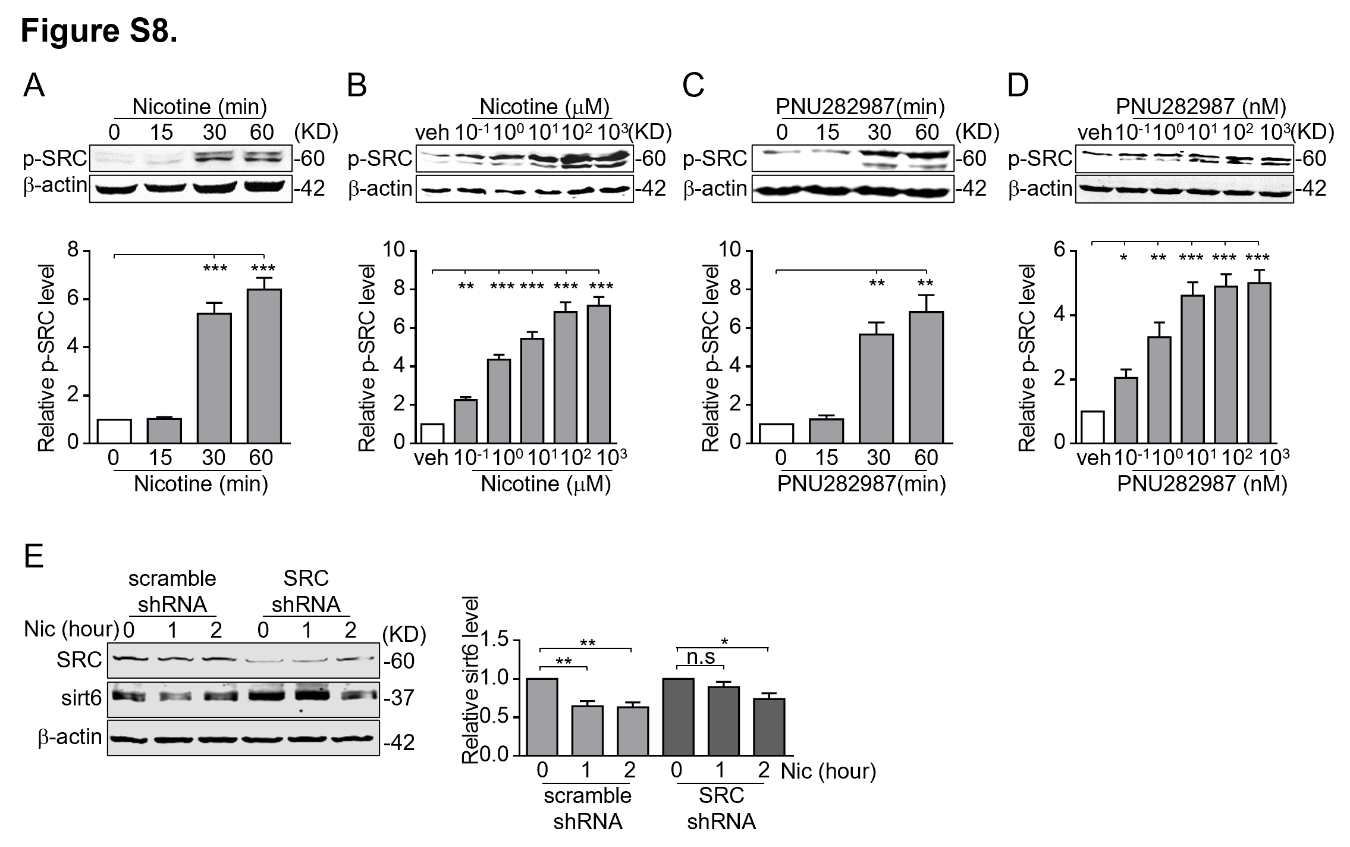


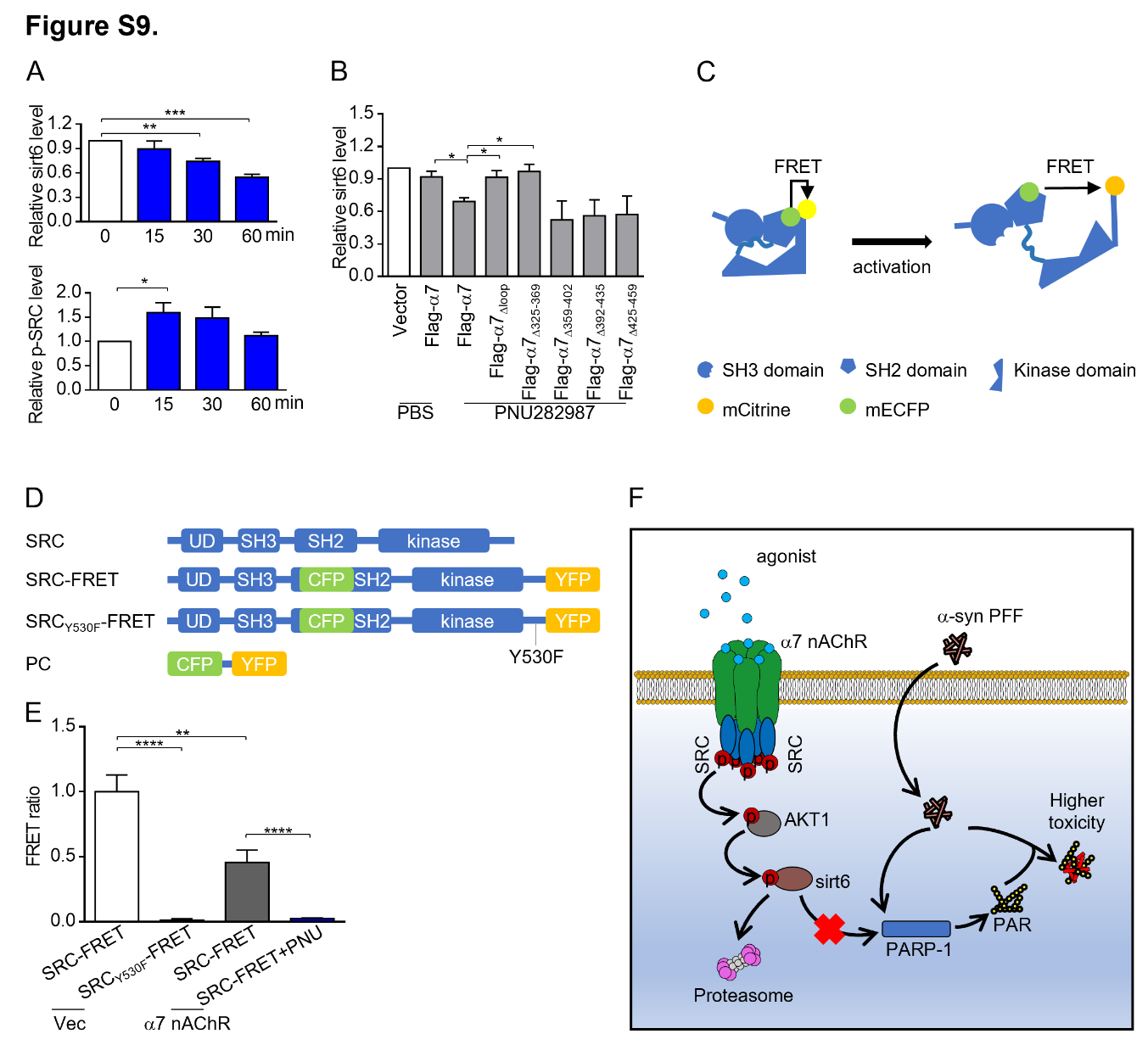

Supplement: pwag004_Supplementary_Data [file pwag004_supplementary_data.docx]
